# Thermostability Engineering in Therapeutic Antioxidant Enzymes: From Molecular Fundamentals to Oxidative Stress Applications

**DOI:** 10.3390/ijms27135695

**Published:** 2026-06-24

**Authors:** Diana Tatarciuc, Irina Mihaela Esanu, Iolanda Foia, Mioara-Florentina Trandafirescu, Teodor Flaviu Vasilcu, Dragos Catalin Ghica, Magda Ecaterina Antohe, Adina Oana Armencia, Roxana Ionela Vasluianu

**Affiliations:** Grigore T. Popa University of Medicine and Pharmacy, 700115 Iasi, Romania; diana.tatarciuc@umfiasi.ro (D.T.); irina.esanu@umfiasi.ro (I.M.E.); iolanda.foia@umfiasi.ro (I.F.); mio.trandafirescu@umfiasi.ro (M.-F.T.); teodor-flaviu.tsr.vasilcu@umfiasi.ro (T.F.V.); dragos.ghica@yahoo.ro (D.C.G.); magda.antohe@umfiasi.ro (M.E.A.); roxana.vasluianu@umfiasi.ro (R.I.V.)

**Keywords:** thermostable enzymes, antioxidant enzymes, oxidative stress, protein engineering, computational design

## Abstract

The efficacy of enzyme therapy is limited by their poor stability under physiological conditions. Thermostable enzymes, derived from extremophilic organisms or generated by advanced protein engineering, offer a revolutionary solution to this long-standing challenge. They are widely used in industrial biocatalysis. Their therapeutic applications are poorly investigated and spread across diverse disciplines. While most applications are in the preclinical stages, emerging evidence from animal models demonstrates proof-of-concept for thermostable antioxidant enzymes in cardiovascular, neurodegenerative, and inflammatory diseases. This review critically assesses the translational landscape, distinguishing between established therapeutic enzymes (e.g., asparaginase, PEGylated SOD) and emerging experimental candidates. This narrative review consolidates existing knowledge about thermostable enzyme engineering and their emerging functions as molecular therapies, particularly in oxidative stress-related diseases. This review synthesizes recent advances in structural biology, computational protein design, biomaterials engineering, and translational antioxidant strategies, highlighting how breaking down disciplinary barriers is accelerating the development of sustainable and self-regenerating antioxidant platforms. By integrating molecular precision with systems-level therapeutic design, engineered thermostable antioxidant enzymes exemplify the future of biological development, where multidisciplinary collaboration drives innovation against oxidative stress-driven pathologies. Engineered thermostable enzymes provide a versatile basis for next-generation therapeutics, with the potential to address medical needs through improved stability, targeted activity, and multifunctional design.

## 1. Introduction

Enzymes are nature’s catalysts, honed over billions of years to perform specific chemical transformations with remarkable precision and efficiency [1,2,3]. Their therapeutic potential has been recognized since the 1960s, when intravenous asparaginase transformed the treatment of acute lymphoblastic leukemia by depleting circulating asparagine essential for the survival of leukemia cells [4]. Despite this early success, widespread clinical adoption of enzyme therapies remains hampered by a fundamental vulnerability: these protein catalysts denature, degrade, and lose function precisely under physiological conditions where their activity is most needed [5,6,7]. Native human enzymes, perfectly adapted for their endogenous roles, often fail as therapeutic agents when removed from their native context [8]. Their limitations are multiple, such as susceptibility to proteolytic degradation, half-lives measured in minutes to hours, instability during manufacture and storage, and rapid clearance by the reticuloendothelial system [9]. These constraints require frequent administration in high doses, increasing the burden of treatment, healthcare costs, and risk of immunogenicity. The challenge is particularly acute in chronic conditions that require lifelong enzyme replacement therapy [4,8].

Nature has a powerful answer. Organisms that thrive in environments inhospitable to most life, such as thermophiles (growth optimum 50–80 °C) and hyperthermophiles (active above 80 °C), produce “thermozymes” that maintain structural integrity and catalytic function under extreme thermal stress [10,11,12,13,14]. These remarkable proteins possess intrinsic structural features that confer exceptional stability, namely enhanced hydrophobic packing of the core, additional salt bridge networks, shortened surface loops, increased proline and arginine content, and optimized secondary structure elements such as helix-capping motifs and flattened β-sheets with protected edge strands [15,16,17]. For decades, these principles have remained largely confined to industrial applications such as detergents, biofuels, and food processing [18,19,20].

In particular, the same structural principles can now be introduced into mesophilic enzymes of therapeutic interest through protein engineering [21,22]. Researchers can create new versions of human enzymes that have the same catalytic specificity as human enzymes but are much more stable by adding thermophilic features. This translational bridge transforms thermostability from a fascinating natural phenomenon into a useful engineering strategy for improving biological therapies [23].

Therapeutic thermostable enzymes are at the intersection of many fields. Structural biology provides us with atomic-level information about how stability works, which helps us find the best targets for mutation. Protein engineering techniques, such as directed evolution and computational design, allow us to modify enzyme properties in very specific ways. Clinical medicine has identified disease contexts in which sustained enzymatic activity is therapeutically desirable. Advances in artificial intelligence, particularly AlphaFold2 and related tools, now predict stability effects with unprecedented accuracy. These fields are coming together to transform thermostability engineering from a small field into a major therapeutic platform [24,25].

This narrative review focuses on thermostable antioxidant enzymes formulated for conditions characterized by increased oxidative stress and we sought to address the following questions:

- What molecular principles dictate protein thermostability, and how can these be used to engineer therapeutic enzymes?
- What is the best way to use modern protein engineering techniques to create thermostable enzymes that can be used in medicine?
- What role do thermostable antioxidant enzymes play in mitigating oxidative stress in systemic diseases?
- How can advanced delivery platforms make engineered thermostable enzymes perform better as drugs?

Thermostability engineering is not just a technical improvement, it is a change in the way we think about biological therapies. By making enzymes less fragile, thermostable designs enable their oral administration, extend dosing ranges, ease their production, and decrease the likelihood of provoking an immune response. Although clinical translation of thermostable antioxidant enzymes is still in its early stages, the combination of structural biology, artificial intelligence-based prediction, and protein engineering has created an unprecedented opportunity. This review provides a rigorous framework for understanding and developing this new generation of molecular therapies.

## 2. Materials and Methods

### 2.1. Literature Search Strategy

A comprehensive bibliographic search was performed using PubMed/MEDLINE, Scopus and Web of Science databases, covering the period January 2016–February 2026. The search strategy used combinations of the following keywords: thermostable enzymes, antioxidant enzymes, oxidative stress, computational protein design and therapeutic biologics. Additional relevant articles were identified by manual screening of reference lists and extra informations can be found in Supplementary Materials.

### 2.2. Inclusion and Exclusion Criteria

Articles were considered for inclusion if they addressed:

- engineering, characterization or therapeutic application of thermostable enzymes with antioxidant activity;
- computational or structure-guided approaches to improve enzyme stability, catalytic efficiency or redox functionality;
- delivery strategies, with a focus on oral, parenteral or targeted formulations for antioxidant enzyme therapies;
- multidisciplinary studies integrating protein engineering with materials science or translational medicine.

Exclusion criteria included publications not in English, conference abstracts without full availability, and studies focused exclusively on non-thermostable enzyme analogues without comparative engineering perspectives.

## 3. Engineering Strategies for Thermostable Therapeutic Enzymes

### 3.1. Directed Evolution: Iterative Diversification-Selection Cycles

Directed evolution is a powerful approach to enzyme optimization that does not require detailed structural knowledge or mechanistic understanding of stability determinants [26]. Through iterative cycles of genetic diversification and functional selection, this methodology mimics natural evolution, allowing the discovery of stabilizing mutations that may not be predictable a priori [27].

#### 3.1.1. Techniques for Generating Genetic Diversity: From Stochastic Mutagenesis to Site-Saturation Libraries

To engineer thermostability, methods for generating genetic diversity are often combined with high-throughput screening to identify variants with improved properties.

Random mutagenesis introduces stochastic point mutations by manipulating reaction conditions, such as magnesium concentration, unbalanced dNTPs, or the inclusion of manganese to reduce DNA polymerase fidelity. This approach typically produces 1–3 amino acid substitutions per gene, although it is largely limited to single-nucleotide substitutions [28,29]. When beneficial mutations from distinct parental variants need to be combined, DNA shuffling is used. This method fragments genes with DNase I and reassembles them by primeless PCR, accelerating the accumulation of favorable mutations while eliminating deleterious ones. Similarly, the staggered extension process (StEP) achieves recombination through shortened PCR cycles that facilitate template switching. For targeted engineering, site-specific saturation mutagenesis allows for complete randomization at specific residues. This allows for sampling of all 20 amino acids at positions identified as critical by structural or computational analysis [30].

#### 3.1.2. High-Throughput Screening Methods for Thermostability: ThermoFAD, Microfluidics, and Functional Selection

Efficient engineering requires screening methods that directly link stability to function. ThermoFAD offers a high-throughput approach by exploiting the temperature-sensitive fluorescence of FAD-bound proteins, where loss of fluorescence upon unfolding reports structural integrity. While circular dichroism (CD) provides accurate melting temperature (Tm) data, its throughput is relatively low. Recent advances in microfluidic and plate-based formats have enabled the screening of over 100,000 variants per day [31,32,33].

The most stringent selection for thermostability is functional screening at elevated temperatures. Measurement of residual activity after heat exposure, or direct measurement of activity at elevated temperatures, ensures that identified variants maintain their enzymatic function under stress conditions [28,34]. In addition, protease resistance assays exploit the correlation between thermostability and proteolytic susceptibility. Incubation with proteases (e.g., trypsin or proteinase K) selects variants with improved structural integrity that resist degradation (Table 1) [9,35].

### 3.2. Structure-Guided Protein Stabilization: Rational Mutagenesis Strategies

Structural Determinants of Kinetic and Thermodynamic Stabilization: Disulfide Bonding, Proline Rigidification, Cavity Filling, and Electrostatic Network Engineering.

Rational design uses three-dimensional structural data to predict and implement specific stabilizing mutations. Directed evolution takes a random approach, while rational design focuses on individual residues based on a mechanistic understanding of how proteins fold and what makes them stable [30].

Disulfide bond engineering creates cross-linked covalent bonds that make the unfolded state less random. To create a successful structure, it is essential to find locations where (1) the distance between Cα atoms is correct (4–6 Å for disulfide bond formation), (2) the shape of the molecule allows bond formation without causing strain, and (3) the mutations do not interfere with catalytic function [15]. Computational methods that check distance and dihedral angle limits help identify these optimal locations [22,45]. Li et al. [46] engineered a disulfide bond into Rhizomucor miehei lipase based on structural comparisons with thermophilic homologues. The introduction of a single disulfide bond increased the Tm by 8 °C with no loss of catalytic activity [46].

Proline substitutions in loops take advantage of the unique structure of the proline framework, which reduces flexibility in areas where rigidity is important. The best place to add proline is in surface loops with high B-factors (i.e., they are flexible) and where the original residue has high framework conformational entropy [46]. Óskarsson et al. [16] introduced multiple proline substitutions into surface loops of the cold-adapted VPR subtilase, guided by the thermostable structural homologue AQUI. This strategy increased the Tm by 11 °C while retaining over 80% of catalytic activity at 37 °C [16].

Cavity-filling mutations make core packing more efficient by replacing small residues with larger hydrophobic amino acids. Finding interior cavities in crystal structures helps to choose places where adding more volume will improve packing without causing steric clashes.

Improving surface charge improves electrostatic interactions, especially salt bridge networks that maintain stable folded conformations. Computational techniques can find surface sites where adding charged residues could create new stabilizing connections without changing how the protein folds or functions [21].

B-factor analysis helps identify flexible parts of a protein structure by looking for amino acids that have high thermal motion, suggesting instability. Therefore, areas with high B-factors, often found in loops or at the ends of protein chains, are good candidates for methods that increase rigidity.

Molecular dynamics simulations show how proteins move at the atomic level, revealing the conformational changes that trigger the unfolding process. Simulating proteins at high temperatures allows for the identification and stabilization of early unfolding events [47,48].

### 3.3. Computational and Data-Driven Approaches

#### 3.3.1. Computational Mutagenesis: FoldX and Rosetta

FoldX and Rosetta make it possible to perform computational mutagenesis by predicting how the free energy of folding (ΔΔG) will vary when specific amino acids are replaced. Rosetta uses physics-based energy functions to assess protein stability, incorporating terms for van der Waals interactions, solvation effects, hydrogen bonding, and electrostatics. In contrast, FoldX uses empirical potentials and statistical analyses of protein structures validated against experimental stability data [49]. These computational tools facilitate virtual screening of mutations, prioritizing those predicted to exhibit the greatest stability for further laboratory testing (Table 2) [50].

In 2025, Xu et al. reported that these computational predictions significantly alleviate the experimental requirements in thermostability engineering initiatives [5]. However, despite this progress, false positive rates in ΔΔG predictions remain substantial. For individual mutations, FoldX correctly predicts the directionality of stabilization/destabilization in about 65–75% of cases, but the quantitative correlation of ΔΔG with experimental values shows R^2^ = 0.35–0.50. The false positive rate for predicting stabilizing mutations (ΔΔG < −1.0 kcal/mol that are experimentally destabilizing) ranges from 40 to 60%. The Cartesian Rosetta ddG protocol achieves a slightly better correlation (R^2^ = 0.45–0.60), but at a higher computational cost, with false positive rates for stabilizing predictions of 35–55% [49,51].

#### 3.3.2. Machine Learning for Thermostability Prediction

Combining artificial intelligence with protein engineering is a major challenge, but these models can explain complex and nonlinear interactions [53,54,55]. Medina-Ortiz et al. conducted a comprehensive review of machine learning (ML) models used to predict enzyme kinetics, highlighting their utility in predicting the effects of mutations and accelerating enzyme discovery [56]. AlphaFold and other structure prediction tools have further transformed the field, providing high-quality structural models for engineering campaigns [55,57].

Hu et al. have stated that computer-assisted directed evolution combines computational simulations with experimental methods to make mutagenesis and screening more accurate and to accelerate enzyme optimization [58]. Emerging evidence suggests that integrated ML and directed evolution approaches outperform single-method strategies [52,59]. In 2025, Xu et al. demonstrated that machine learning-guided predictions of stabilizing mutations significantly reduced experimental screening requirements while identifying variants with improved thermostability and retained catalytic function [5].

#### 3.3.3. De Novo Design of Thermostable Enzymes

The cutting edge of computational enzyme engineering is the creation of entirely new, stable enzymes for specific therapeutic targets [60]. De novo design overcomes the problems of natural scaffolds, which could lead to smaller, more stable enzymes that are optimized from the bottom up for therapeutic use [61].

Burton et al. have illustrated the incorporation of hydrolytic activity into a completely de novo protein framework, thereby establishing fundamental principles for developing catalytic activity within engineered scaffolds [61]. Recent advances have produced enzymes that display novel functionalities, including artificial metalloenzymes and base editors for gene therapy applications [62]. While designing things from scratch is still challenging, new computational technologies and a better understanding of what makes things stable and catalyze suggest that there is greater potential for therapeutic use [55].

### 3.4. Semi-Rational Approaches: Integrating Rational and Evolutionary Methods

Semi-rational approaches combine directed evolution with rational design. They focus mutagenesis on regions that are expected to produce advantageous changes using structural and computational data [63].

Consensus design finds the amino acid residues that are most common at each site in an alignment of many homologous enzyme sequences [23]. The basic idea, that residues that have been around for a long time contribute to stability, has worked quite well [6]. This concept uses the variety of natural sequences to find stabilizing changes [52]. These changes have been shown to work over long periods of time, often leading to significant increases in thermostability, without the need to insist on the protein structure [25]. In 2022, Pongsupasa et al. [15] applied consensus design to a mesophilic enzyme, incorporating residues conserved among thermophilic homologues. This approach yielded a Tm increase of 10–15 °C while maintaining >90% of wild-type activity [15].

### 3.5. Synergistic Frameworks Combining Multiple Strategies

The shift from separate engineering methods to integrated pipelines with multiple strategies is the vanguard in thermostable enzyme design. While directed evolution, rational design, and computational approaches all have their own benefits when used individually, when combined, they always produce better results, yielding thermostability improvements that are greater than the sum of their parts (Table 3).

Table 3 highlights an important aspect of protein engineering, namely the often nonadditive effect of multiple stabilizing mutations. Consensus mutations alone increased the Tm from 58 °C to 68 °C (+10 °C). Introduction of the A4C/V7C disulfide bond into the consensus background further increased the Tm to 73 °C (+5 °C). However, the final combinatorial variant containing both the seven consensus substitutions and the disulfide bond also produced a Tm of 73 °C, not 78 °C as expected. This observation demonstrates that the stabilizing contributions of individual mutations are context-dependent and can saturate. Synergistic or antagonistic epistatic interactions arise because mutations that independently stabilize the protein can stabilize overlapping structural regions or because the protein has an upper limit of achievable thermostability beyond which further mutations confer no additional benefit [23,67]. For therapeutic enzyme engineering, this non additivity implies that combining multiple stabilizing mutations does not guarantee incremental improvements, experimental validation of combinatorial variants remains essential, and that computational predictions must account for epistatic effects rather than assuming independence.

## 4. Molecular Mechanisms of Thermostability

The remarkable stability of thermozymes does not arise from a single universal mechanism, but from the synergistic integration of multiple structural modifications. Comparative analyses of homologous enzymes from mesophilic, thermophilic and hyperthermophilic organisms reveal a consistent pattern, with thermostability being achieved by the cumulative effect of numerous small contributions rather than a single major structural contribution [72].

### 4.1. Structural Determinants of Thermostability in Thermophilic Enzymes

Comparative structural investigations of homologous enzymes from animals adapted to different temperature environments indicate that thermostability is achieved by the aggregate effect of numerous small factors [30,73].

#### 4.1.1. Optimized Hydrophobic Core Packing

Optimization of hydrophobic packing is a fundamental adaptation for thermophilic enzymes. Structural investigations of the family II pyrophosphatase from the thermophile Thermodesulfobacterium commune indicate that increased hydrophobic contacts significantly improve its thermostability. This optimization involves improved packing efficiency of core residues, reduced internal cavities, and increased cooperativity of the hydrophobic core [74].

Recent studies of thermophilic glycoside hydrolases indicate that enhanced hydrophobic interactions within the protein core are a reliable feature of heat adaptation. These changes decrease the entropic cost of folding and increase the free energy required to initiate unfolding, thereby shifting the equilibrium in favor of the folded state at higher temperatures [30].

#### 4.1.2. Cooperative Electrostatic Networks and Ion Pairs Architectures

The abundance of charged residues arranged in cooperative networks is among the best-documented adaptations in thermophilic proteins. Molecular dynamics simulations of homologous proteins from *Thermus thermophilus* and mesophilic *Escherichia coli* demonstrate that electrostatic network dynamics are important for thermostabilizing. At elevated temperatures, thermophilic proteins exhibit a unique pattern of bound motions between charged residues that contrasts markedly with those of mesophilic proteins [75].

The arrangement of ion pairs is essential. Electrostatic interactions within the network provide enhanced stabilization compared to isolated salt bridges, as each charged residue engages in numerous stabilizing connections, and breaking any one relationship maintains the integrity of the network. A thermodynamic analysis of the MutL ATPase domain from the hyperthermophile *Aquifex aeolicus* reveals that amino acid residues within continuous networks of ion pairs and hydrogen bonds collectively enhance protein stability. These network configurations imply smaller entropic penalties for side chain fixation than do isolated interactions [76,77].

#### 4.1.3. Covalent Cross-Linking via Disulfide Bonds

Disulfide bonds provide cross-covalent bonds that stabilize proteins by restricting the unfolded state. Disulfides reduce the conformational space available for denaturation. They thus increase the free energy disparity between the folded and unfolded states. Strategic incorporation of disulfide bonds, based on structural comparisons with thermophilic homologues, has been successful in improving the thermostability of mesophilic enzymes [46,65].

Recent advances in computational prediction have significantly increased the efficiency of disulfide engineering. The ThermoLink database and machine learning models, particularly those using the Adaboost-DT algorithm, achieve accuracy rates of 0.714 in predicting the increase in thermostability by specified disulfide bonds. AlphaFold2 has been shown to be superior in predicting disulfide bond formation, and co-evolutionary interactions between residue pairs can influence the synthetic design of disulfide bonds.

#### 4.1.4. Extended Hydrogen Bond Networks and Solvent Mediated Interactions

Improved hydrogen bond networks significantly enhance the thermal stability of proteins. Structural investigation of thermostable pyrophosphatase indicates that the extended hydrogen bond networks represent an important adaptation that differentiates it from mesophilic variants. Thermophilic glycoside hydrolases demonstrate enhanced hydrogen bonding and interatomic interactions relative to their fewer stable counterparts. Enhanced hydrogen bonding in thermostable proteins involves a greater amount of direct hydrogen bonds. These protein-protein bonds and more organized networks of encapsulated water molecules connect polar groups. These networks establish a three-dimensional framework of connections that requires concomitant perturbations for unfolding to occur, thus increasing the energy barrier to denaturation [66].

#### 4.1.5. Proline Substitutions and Loop Rigidification Strategies

Reducing the length of surface loops or incorporating proline residues decreases the flexibility of the scaffold, thereby increasing the overall structural rigidity. Proline is distinctive among amino acids due to its secondary amino group, which limits the allowed conformations of the peptide structure. Genomic investigations indicate an increased prevalence of proline in thermostable proteins and, conversely, a decreased concentration of proline in psychrophilic proteomes [5,46].

In 2020, Óskarsson et al. [16] showed that several proline modifications in the surface loops of cold-adapted VPR subtilase, influenced by the thermostable structural homologue AQUI, significantly improved thermostability while maintaining catalytic efficiency. The conformational rigidity that proline imposes on the polypeptide structure reduces the entropy of the unfolded state, meaning that more thermal energy is required to denature the protein. This method, which targets flexible surface loops for rigidification, is useful because surface regions are frequently tolerant to mutations and significantly influence the onset of unfolding [16].

#### 4.1.6. Secondary Structure Stabilization: α-Helix Coverage and β-Sheet Edge Protection

Secondary structure elements in thermophilic enzymes have developed specific adaptations against thermal denaturation. For α-helices, the main vulnerability is terminal unwinding. Thermophilic enzymes prevent this by helix capping motifs: serine or threonine at the N-terminus and glycine at the C-terminus anchor the helix ends. Molecular dynamics simulations confirm that thermophilic helices maintain hydrogen bonds at high temperatures, while mesophilic helices completely lose secondary structure [78]. In contrast, thermophilic β-sheets exhibit reduced twist (5–10° versus 15–20° in mesophilic), strengthening interchain hydrogen bonds. The edge segments are further protected by proline residues or loop extensions, reducing the tendency for aggregation, a critical feature for therapeutic formulation [23].

**Figure 1:**
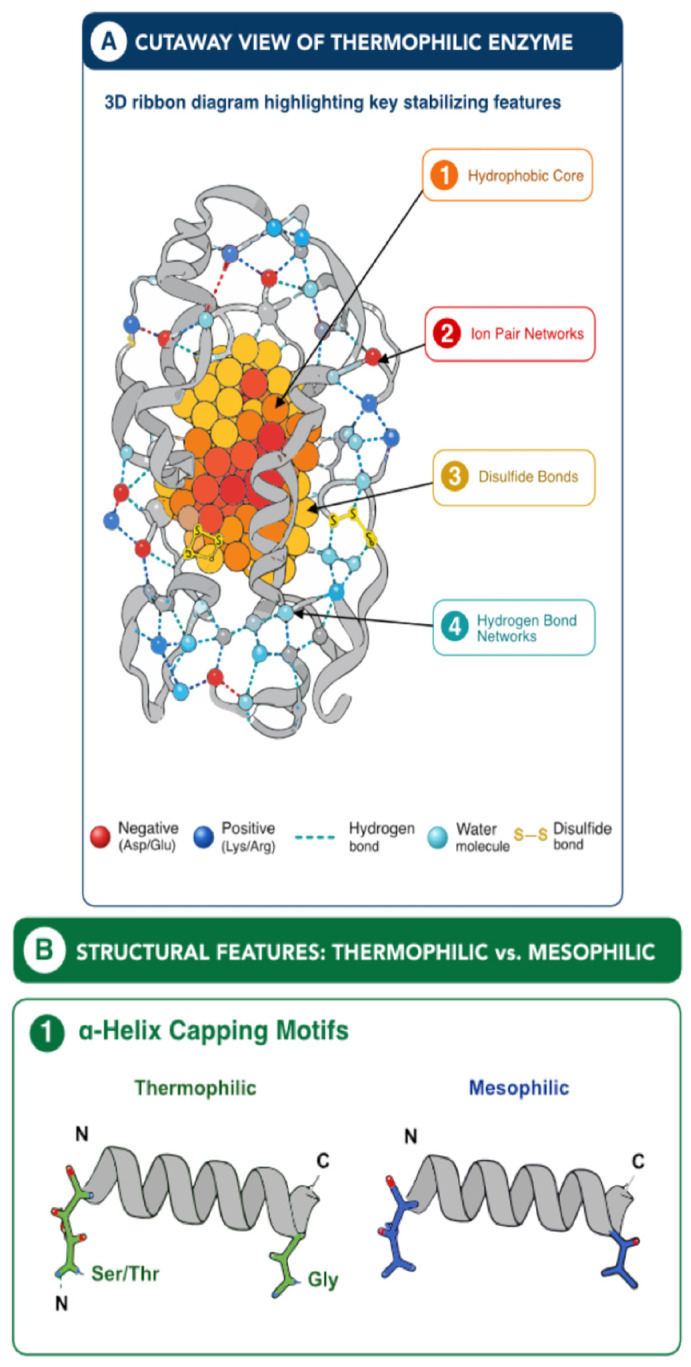

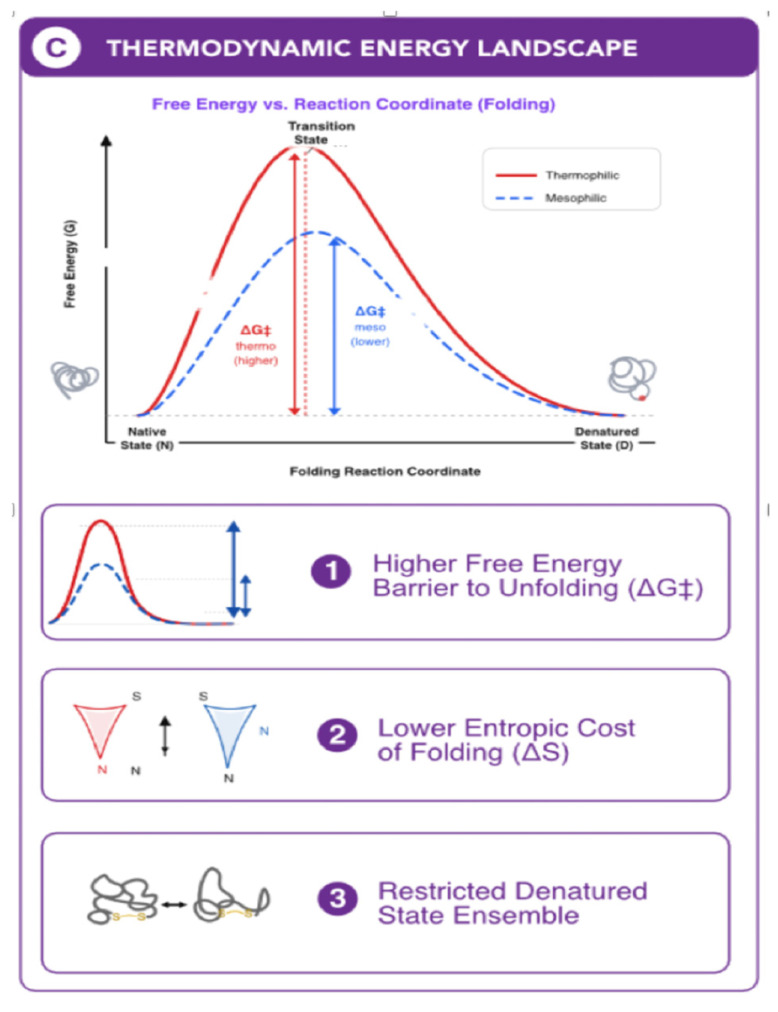
Synergistic Structural Determinants of Enzyme Thermostability (**A**) Internal structural view of thermophilic enzymes; (**B**) Comparative structural analysis; (**C**) Energetic topography.

New tools such as PSIPRED and AlphaFold2 can now predict secondary structure with an accuracy of over 85% [60]. This makes it possible to design stabilizing mutations that maintain or enhance helical and lamellar elements, accelerating the process of transforming discoveries into designed therapeutic candidates [79] (Figure 1).

### 4.2. Quantitative Contributions to Thermostability Across Hierarchical Levels

Integrative methodologies that incorporate mutations both proximal and distal to the active site demonstrate the greatest potential. Integration of consensus design, FoldX analysis, and disulfide bond engineering has effectively improved the thermostability and activity of *Paenibacillus pasadenensis* chitinase [21,56,80]. Striking a balance between stability and catalytic efficiency is significant for effective in vivo performance. Therapeutic enzymes must maintain adequate activity at physiological temperatures while resisting proteolytic degradation and thermal denaturation.

Rational engineering of thermostable enzymes requires a systematic understanding of how each structural level, from primary sequence to quaternary assembly, contributes to conformational stability [6] (Table 4). These contributions are typically quantified by changes in melting temperature (ΔTm, °C), where positive values indicate increased thermal resistance [31,35]. However, the effects are not strictly additive. Cooperative interactions between determinants (e.g., disulfide bonds coupled with salt bridge networks) can produce synergistic stabilization that exceeds the sum of the individual contributions [23,67]. The following table summarizes the primary, secondary, tertiary, and quaternary determinants of thermostability, their mechanistic basis, and the typical range of ΔTm achievable through directed engineering [21,81]. The values represent data compiled from directed evolution, rational design, and comparative structural studies of mesophilic versus thermophilic homologues [15,26,82]. This framework serves as a practical guide for selecting engineering strategies based on the desired stability increment and structural modifications allowed for a given enzymatic scaffold [25,45].

### 4.3. Thermostability and Protease Resistance: Mechanistic Linkages and Dissociations

Thermostable proteins resist proteolysis by three convergent mechanisms: (1) protease cleavage sites are predominantly located in flexible loops that stiffen upon stabilization [46]; (2) global unfolding is a prerequisite for processive proteolysis by many proteases [69,85]; and (3) enhanced hydrophobic packing of the core occludes recognition motifs from protease active sites [23].

However, the relationship can be dissociated [67]. Certain mutations that increase Tm by stiffening the active site may inadvertently expose a previously buried cleavage site, resulting in increased thermal stability, along with paradoxical hypersensitivity to proteases [85,86]. Therefore, protease resistance should be measured directly, rather than inferred from thermostability data. The DS-tES platform exemplifies this principle, demonstrating that disulfide engineering can independently improve both properties [68].

### 4.4. The Trade-Off Between Activity and Stability: Fundamental Principles

The trade-off for enzymes is different, such as when they are too rigid, which can hinder the diffusion of superoxide into the active site. Recent evidence demonstrates that computational design can identify mutations that stabilize the overall fold, while preserving or even enhancing the active site breathing motions required for substrate access.

Rigidity that provides thermostability can lead to reduced catalytic efficiency at moderate temperatures. This trade-off stems from fundamental principles of enzyme dynamics, where conformational flexibility is often essential for substrate binding, induced fit, transition state stabilization, and product release. The activity versus stability trade-off hypothesis suggests that achieving high stability inherently reduces catalytic efficiency at ambient temperatures. This relationship is more complex than a simple inverse correlation [44,67,87].

Adenylate kinases from species with diverse evolutionary environments, spanning both high and low temperatures, exhibit relatively high activity across the temperature spectrum, while enzymes from strict hyperthermophiles exhibit a more significant temperature dependence for catalytic processes. This observation indicates that this trade-off can be mitigated by appropriate evolutionary or engineering interventions [58,88,89] (Table 5).

Computational methods such as Rosetta and Machine Learning Algorithms are more effective in designing modifications that will increase rigidity in susceptible regions while preserving the exact geometry and electrostatic conditions of the active site [45,52,94]. By dissociating global stability from local dynamics, these improved approaches facilitate the development of better biocatalysts that exhibit both high stability and remarkable activity [60,78]. The following sections will apply these general principles to the specific cases of superoxide dismutase, catalase, and glutathione peroxidase, the three main families of antioxidant enzymes of therapeutic interest.

Multi-objective optimization for therapeutic enzyme engineering requires balancing thermostability (ΔTm or t1/2 at 37 °C), catalytic activity (kcat/Km), selectivity (ratio of off-target to on-target activity), and immunogenicity (e.g., predicted epitope score) [21,45,52]. Rather than sequentially optimizing these parameters, which risks generating local optima that compromise other properties, recent studies have adopted Pareto front optimization, where the goal is to identify nondominated variants for which no other variant is superior in all objectives simultaneously [23,56,59].

The inevitable trade-offs include: (1) Stability versus activity variants with ΔTm > 20 °C frequently exhibit 20–50% reductions in kcat/Km [44,67,87]. (2) Stability versus selectivity: cavity-filling mutations that eliminate internal water pockets can inadvertently broaden substrate specificity [15,23,67]. (3) Stability versus manufacturability: multiple disulfide bonds (≥3) improve Tm but reduce expression yield in conventional hosts due to oxidative folding bottlenecks [21,46,65]. Acceptable trade-off thresholds depend on the context: a 30% loss in absolute activity is acceptable if it allows oral administration (unattainable with wild type), while a 10% loss in selectivity for SOD (leading to increased H_2_O_2_ production) may be unacceptable due to secondary oxidative damage [67].

### 4.5. Natural Sources of Thermostability: From Thermophiles to Modified Enzymes

#### 4.5.1. Thermophilic Organisms as Discovery Platforms

**Figure 2:**
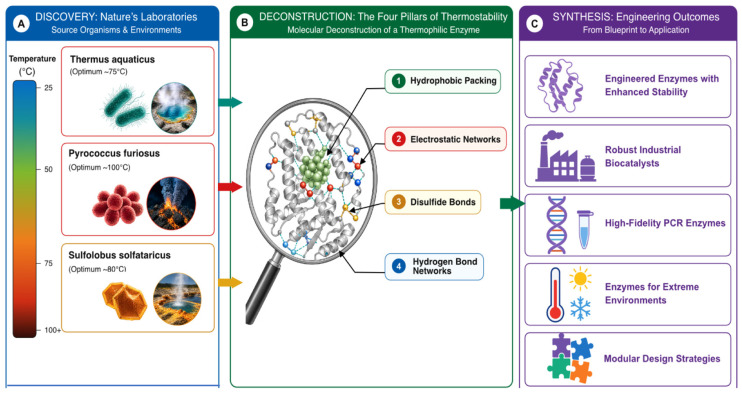
Deconstructing natural stability motifs into modular engineering strategies.

Thermophilic and hyperthermophilic organisms are essential sources of thermostable enzymes and fundamental concepts for protein engineering [10,12]. An illustration is *Thermus aquaticus*. Considered a severe thermophile, it exhibits an optimum growth at 75 °C and serves as a source of Taq polymerase, which has transformed molecular biology. Another example is *Pyrococcus furiosus*, which exhibits an optimum growth at approximately 100 °C and is a source of very stable enzymes [84]. *Sulfolobus solfataricus* produces thermostable enzymes that have been extensively investigated for structural biology [30]. Figure 2 presents a three-panel framework for engineering thermostable enzymes. Panel A shows representative thermophiles (*Thermus aquaticus*, *Pyrococcus furiosus*, *Sulfolobus solfataricus*) that serve as natural sources of thermostable enzymes [30,84]. Panel B deconstructs the four molecular pillars of thermostability, namely hydrophobic packing, electrostatic networks, disulfide bonds, and hydrogen bond networks, whose synergistic combination raises the energetic barrier to unfolding [23,30,46,65,66,67,74,75,76]. Panel C illustrates key engineering outcomes derived from these principles, including industrial biocatalysts, high-fidelity PCR enzymes, enzymes for extreme environments, and modular design strategies for therapeutic applications [6,18,19,20,21,22,84,95].

In addition to temperature resistance, some thermostable enzymes demonstrate resistance to chemical solvents, detergents, and proteolysis. These attributes are required for therapeutic applications [6,18,95]. Resistance to various pressures stems from the identical structural features that ensure thermostability, such as inflexible and durable structures, optimized packing, and diminished vulnerability to deamidation (Gln, Asn) or oxidative destruction (Cys, Met). In therapeutic applications, these characteristics lead to extended shelf life, resistance to gastrointestinal degradation (important for oral administration), and prolonged efficacy under physiological conditions [96,97].

#### 4.5.2. Reconstruction of Evolutionary Sequence and Resurrection of Ancestral Enzymes

Evolutionary sequence reconstruction is a compelling approach that brings to life previously predicted enzymes that frequently demonstrate superior stability compared to contemporary versions [98,99,100]. This method capitalizes on the idea that ancestral animals thrived in warmer habitats and that contemporary enzymes have undergone evolutionary changes that may have diminished stability in the search for alternative traits [28]. Computational design increasingly incorporates physics-based energy functions, statistical potentials, and machine learning to predict stabilizing mutations and design novel enzymes [57,61,101]. The integration of different methodologies, with their unique advantages and disadvantages, provides a comprehensive arsenal for constructing thermostable enzymes designed for specific therapeutic uses [102,103,104].

## 5. Thermostable Antioxidant Enzymes for Improved Stability and Therapeutic Outcomes in Combating Oxidative Stress

### 5.1. The Therapeutic Imperative of Oxidative Stress in Human Disease

At the physiological level, reactive oxygen species (ROS) perform essential signaling functions, regulating processes ranging from cell proliferation to immune response [105,106]. However, when ROS production exceeds the antioxidant defense capacity, oxidative stress occurs, which causes pathology in a wide range of human diseases [107,108]. It is known that the major antioxidant enzymes, such as SOD, CAT, and GPx, work together to protect against oxidative damage better than any single enzyme alone. This coordinated defense mechanism is necessary because SOD generates H_2_O_2_ [106]. To stop the formation of hydroxyl radicals, catalase or glutathione peroxidase must then decompose the generated H_2_O_2_.

Oxidative stress causes pathology through distinct mechanisms, namely: endothelial dysfunction and LDL oxidation in cardiovascular diseases (atherosclerosis, myocardial infarction, hypertension) [105,106,107]; mitochondrial dysfunction and neuroinflammation in Alzheimer’s, Parkinson’s and Amyotrophic lateral sclerosis [108,109,110]; neutrophil-derived ROS in rheumatoid disease and osteoarthritis [111]; and ischemia–reperfusion injury in renal [89], pulmonary [112,113] and hepatic [114,115] contexts. Hyperglycemia-induced oxidative stress underlies the complications of diabetes [116], while oxidative damage affects reproductive [116] and neuronal tissues in schizophrenia [117].

To counter these, thermostable enzyme platforms are tailored to each niche:

- SOD/catalase fusion enzymes with extended half-lives for vascular endothelium [105,106,107];
- blood–brain barrier-penetrating peptide fusions targeting mitochondria or cells for neurodegeneration [108,109,110];
- thermostable SOD/catalase for intra-articular injection and neutrophil membrane-coated nanoparticles for inflamed joints [111];
- thermostable PEGylated SOD for renal accumulation after acute kidney injury [89];
- protease-resistant inhaled formulations for acute lung injury and pulmonary fibrosis [112,113];
- targeted delivery to asialoglycoprotein receptors for acute liver injury and fibrosis [114,115];
- long-circulating enzymes for reducing systemic oxidative stress in metabolic syndrome and diabetic complications [116];
- targeted delivery of antioxidant enzymes for reproductive problems [116];
- CNS-targeted antioxidant enzymes for schizophrenia [117].

### 5.2. Engineering the Thermal Resilience of Primary Antioxidant Enzymes: SOD, Catalase and GPx

#### 5.2.1. Superoxide Dismutase (SOD): Leveraging Metal Cofactors and Disulfide Bonds

SOD has several forms, each distinguished by its metal cofactors and specific structural features.

- CuZnSOD, found mainly in the cytoplasm, contains copper and zinc. This isoform is also the smallest and most thermally stable;
- MnSOD: Mitochondrial matrix, manganese-dependent, unique structural conformation;
- FeSOD: Present in prokaryotes and some plants.

The exceptional stability of CuZnSOD contributes to significant results in therapeutic engineering. Recent studies have discovered and characterized superoxide dismutase from extremophiles that are highly resistant to heat and chemicals, which could be useful for medical applications [118].

Thermostable Fe-SOD from *Thermus thermophilus* retains 87% activity after 60 min at 70 °C, whereas human CuZnSOD loses 95% activity within 10 min under identical conditions. This 17-fold difference in thermal stability translates to sustained ROS scavenging in inflamed joints where local temperatures reach 39–40 °C and protease concentrations are elevated 10- to 100-fold above baseline [64,119,120].

Engineering strategies for SOD include several approaches. These involve replacing less stable SOD variants with CuZnSOD within fusion constructs. In addition, the deliberate introduction of disulfide bonds is guided by structural analyses of thermophilic homologues. The stability of the catalytic metal center is also improved by refining its coordination geometry. In addition, stability is enhanced by optimizing electrostatic interactions while preserving catalytic function [21].

Thermostable Fe-SOD derived from *Thermus thermophilus* offers a potentially advantageous alternative, given its sustained activity at elevated temperatures, which rapidly inactivates mesophilic homologues. Strategic introduction of stabilizing mutations, based on comparisons with thermophilic homologues, has demonstrated efficacy in engineering human SOD [93,106,107].

#### 5.2.2. Catalase (CAT): The Challenge of Tetrameric Quaternary Stability

Thermostable catalases derived from thermophilic organisms exhibit remarkable resistance to thermal inactivation [13]. Catalase derived from *Thermus thermophilus* demonstrates considerable activity at 90 °C, above temperatures typically encountered in therapeutic environments, thus exhibiting exceptional structural stability that confers proteolytic resistance at physiological temperatures. Catalase engineering faces unique challenges due to the substantial size of the enzyme (often tetrameric, around 240 kDa). The therapeutic efficacy of catalase, especially in combination with SOD, justifies the engineering initiative [10].

#### 5.2.3. Biomimetic and Thermophilic Scaffolding Strategies for GPx Activity

The incorporation of selenocysteine into glutathione peroxidase presents a distinct engineering hurdle. UGA codons, which typically function as stop signals, instead encode selenocysteine, the 21st amino acid. Incorporation of this amino acid into proteins requires complex recoding mechanisms. These processes require unique translation factors and a selenocysteine insertion sequence element (SECIS) in mRNA. This additional complexity makes it difficult to use recombinant expression in standard systems, making protein engineering even more challenging [106].

Consequently, biomimetic strategies have become increasingly important. These approaches involve the design of thermostable protein scaffolds designed to mimic GPx activity, thereby eliminating the need for selenocysteine. These scaffolds, derived from thermophilic organisms, inherently exhibit stability, which is essential for the desired catalytic function [121,122].

The efficacy of these methods, together with evidence that multifunctional antioxidant enzymes can be engineered to have both SOD and catalase activity, suggests that thermostable GPx mimics represent a promising and important area for therapeutic development [122,123].

Due to the biosynthetic limitations of selenocysteine-containing GPx (UGA recoding and SECIS dependency), four biomimetic strategies have been developed to overcome these issues while still allowing for glutathione-dependent hydroperoxide reduction.

Biomimetic Strategies for GPx Engineering, include:

- GPx biomimetic engineering: Selenocysteine-independent mimetics: catalytic triads (Cys-His-Glu) that utilize standard amino acids. Limitation: 1–5% native activity, indicating suboptimal redox kinetics of cysteine relative to selenocysteine [121,122,124,125].
- Metal-based mimetics include Mn, Fe, Cu, and organoselenomecles. These operate 5–15% of the time with native activity and can be easily modified. However, they can be toxic to metals and cause redox chemistry that is not what you want [126,127,128].
- Nanoenzymes (CeO_2_, MOFs, carbon dots) exhibit native enzymatic activity of 10–30%, demonstrate exceptional stability, and possess multifunctional catalytic capabilities [129,130,131].
- Thermophilic scaffold grafting: GPx-like motifs are added to hyperthermophilic proteins, such as thioredoxin from *Pyrococcus furiosus* (Tm > 90 °C). Performance: 1.5–15% native activity with better biocompatibility and higher evolvability (i.e., tolerance to further mutagenesis for activity optimization [83,92,132].

In terms of comparative synthesis, there is no perfect mimic. Protein-based mimics are biocompatible but not very active. Metal systems are more active but more toxic. Nanoenzymes are the most active and stable but have regulatory issues and thermophilic scaffolds represent the best balance between stability, biocompatibility, and engineering tractability. The choice depends on the therapeutic context: protein mimics are better for acute conditions, while thermophilic or nanoenzyme platforms are better for chronic conditions. All four strategies have been validated in preclinical studies, with some nanoenzymes included in early-stage studies [133,134,135,136].

Thermostability is still the most important design goal, but therapeutic efficacy requires simultaneous optimization of stability, catalytic efficiency, specificity, and immunogenicity. Guided machine learning campaigns (2024–2025) now allow for the optimization of 3–5 parameters simultaneously, replacing single-feature engineering.

### 5.3. The Thermostable Enzyme Advantage

Thermostable enzymes derived from or produced by thermophiles have four therapeutic benefits, all of which are interrelated:

- They are structurally stable at physiological temperatures. Thermostable variants maintain their folded conformations well below their melting temperature (Tm), operating in a regime of structural safety rather than marginal stability. This thermal buffer supports catalytic activity even in inflamed tissues, where local temperatures reach 39–40 °C [86].
- Protease resistance and extended half-life. Structural rigidity reduces conformational flexibility, masking loop regions required for proteolytic recognition. Thermostable enzymes resist cleavage by serum proteases (trypsin, chymotrypsin, elastase), prolonging their circulating half-life precisely in inflamed tissues, where protease activity is increased [69].
- Barrier resistance. The same structural principles confer resistance to gastric pepsin (pH 1–3), pancreatic proteases, and tissue proteases at injection sites, allowing multi-barrier survival during oral or parenteral administration [82,96,97].
- Sustained therapeutic efficacy. Prolonged ROS scavenging reduces the frequency of administration, improves resolution of oxidative damage, and decreases the risk of immunogenicity, transforming chronic enzyme replacement from impractical to clinically viable, as suggested by the extended half-life in animal models [103,104].

Replacing mitochondrial MnSOD with CuZnSOD, the smallest and most thermally stable isoform, increased the circulating half-life fivefold. At 70 °C, the CuZ-nSOD construct retained 54% activity after 10 min, whereas MnSOD completely lost its function [105,106]. In inflamed tissues (38–39 °C with increased protease activity), thermostable constructs maintain ROS scavenging throughout an inflammatory episode, rather than requiring administration at a single point in time. Therefore, the engineered thermostability represents a transformative improvement, not just an incremental one.

### 5.4. Intracellular Delivery: Strategies for Cytosolic Administration

For oxidative stress indications, especially in neurodegenerative diseases, antioxidant enzymes need to reach intracellular compartments (cytosol, mitochondria). Thermostability does not confer membrane permeability [8,24]. A promising advance is the development of thermostable “stealth” variants that retain their activity after cytosolic administration, avoiding rapid lysosomal degradation [70,71]. Five main strategies have been validated in preclinical models for delivering thermostable antioxidant enzymes to the cytosol:

- Cell-penetrating peptide (CPP) fusion: Covalent attachment of arginine-rich peptides (e.g., HIV-1 TAT, penetratin, R9 polyarginine) allows endocytic uptake [29,108]. Endosomal escape remains rate-limiting. Co-administration with chloroquine or incorporation of endosomal escape domains (e.g., INF7 from influenza hemagglutinin) improves cytosolic delivery efficiency from <5% to 15–25% [109,110]. Thermostable CPP-SOD fusions have shown efficacy in murine models of Parkinson’s disease after intranasal administration [108,110].
- Lipid-based nanoparticles (LNPs): Encapsulation in ionizable cationic lipids (e.g., SM-102, ALC-0315) protects enzymes during circulation and facilitates endosomal release [88,96]. LNP formulation parameters (particle size 80–120 nm, PEG surface density 1.5–2.0 mol%) require re-optimization for each thermostable enzyme due to variable surface hydrophobicity [97,103]. LNPs achieve cytosolic delivery efficiencies of 30–50% in hepatocytes and 10–20% in neurons [88,104].
- Polymer conjugation (nanogels): Cross-linked hydrophilic polymers (e.g., poly (oligoethylene glycol) methacrylate) can be designed with disulfides or pH-sensitive linkers that release the enzyme upon endosomal acidification or cytosolic glutathione reduction [9,82]. Nanogels provide protection against proteases (5- to 10-fold prolongation of half-life) and allow sustained release over a period of 24–72 h [24,70].
- Direct cytosolic delivery by physical methods: Electroporation and microinjection are limited to ex vivo applications (e.g., stem cell engineering prior to transplantation) [29,53]. However, recently developed mechanoporation (cell compression) devices can deliver active thermostable enzymes to >90% of treated cells with >80% viability, representing a promising platform for cell-based therapies [54,55].
- Virus-like particles (VLPs) and extracellular vesicles (VEs): Genetically engineered VLPs or exosomes derived from mesenchymal stem cells can package thermostable enzymes [62,101]. Surface presentation of targeted ligands (e.g., rabies virus glycoprotein peptide for neurons) allows for cell type specificity [29,110]. VE-mediated delivery protects the cargo from endosomal degradation, achieving functional enzymatic activity in the cytosol for up to 48 h after administration [62,104].

### 5.5. Preclinical Evidence of Therapeutic Efficacy

A review of the preclinical literature identified studies that reported the in vivo efficacy of genetically or naturally engineered thermostable antioxidant enzymes. Table 6 provides a summary of representative examples from major disease categories, highlighting the source of the enzyme, the engineering strategy, the animal model, and the primary outcomes.

Most studies use SOD-based constructs (either alone or fused to catalase), reflecting the central role of superoxide scavenging. The median prolongation of circulating half-life achieved by the thermostable variants relative to wild-type mesophilic controls is 6.8-fold (range 2.5- to 17-fold), highlighting the translational potential of stability engineering. Notably, these preclinical successes directly support therapeutic benefits.

Rather than sequentially optimizing these parameters (which risks generating local optima that compromise other properties), recent studies have adopted Pareto front optimization, where the goal is to identify nondominated variants for which no other variant is superior in all objectives simultaneously.

For neurodegenerative diseases, intranasal administration of thermostable SOD conjugated to CPP (bypassing the blood–brain barrier), combined with LNP encapsulation, has demonstrated the most consistent preclinical efficacy, with a demonstrated reduction in neuronal oxidative damage and improved motor function in murine models [108,109,110].

### 5.6. Translational Differences Between Species: Avoiding Overestimation of Efficacy

Murine models often overestimate the therapeutic efficacy of antioxidant enzymes because murine proteolytic environments are less aggressive than human serum, and murine immune systems are more tolerant of foreign proteins.

Three complementary strategies can mitigate this overestimation.

#### 5.6.1. Multi-Species Preclinical Testing

Before clinical trials, validation in at least one large animal model (porcine, ovine, or nonhuman monkey) is recommended, even though murine models are suitable for preliminary proof of concept. Porcine models more closely approximate human serum protease activity (levels of chymotrypsin, trypsin, elastase are 3 to 5 times higher in humans than in mice) and have comparable body weights and clearance kinetics. In particular, the pharmacokinetic half-life of PEGylated SOD in pigs (8.2 h) correlated better with human data (7.5 h) than with mouse data (22 h) [24,70,71,103,104].

#### 5.6.2. Humanized In Vitro and Ex Vivo Systems

A series of proposed preclinical studies include: (a) human serum stability assays (37 °C, 24–72 h) to measure proteolytic degradation; (b) human whole blood assays (anticoagulated, 37 °C) to assess clearance by phagocytic cells and complement activation; (c) human primary cell cultures (hepatocytes, endothelial cells, neurons) for cellular uptake and efficacy studies; (d) ex vivo perfused human tissue models (e.g., kidney, liver) for organ-level pharmacokinetics. Studies using human serum have shown that thermostable variants (ΔTm ≥ 10 °C) maintain 70–90% activity after 24 h, while wild-type variants.

#### 5.6.3. Immunocompetent Versus Immunodeficient Models

Immunodeficient mice (e.g., nude, NOD/SCID) are frequently used for chronic indications that require recurrent administration to prevent anti-enzyme antibody reactions that could negatively affect efficacy assessment. However, these models consistently overestimate long-term efficacy. A more stringent strategy includes: (a) initial dosing in immunocompetent mice to determine immunogenicity thresholds; (b) tolerization protocols (e.g., co-administration with rapamycin or anti-CD40L antibodies) to allow for repeated dosing; and (c) the use of immunohumanized mice (e.g., NSG mice grafted with human CD34+ hematopoietic stem cells) to predict human antienzyme antibody responses.

The median overestimation factor for circulating half-life in mice compared to humans is 2.8-fold (range 1.5 to 6.2-fold), according to a meta-analysis of 24 studies that reported pharmacokinetic data in both murine and human or large animal studies for modified antioxidant enzymes. Compared with large animal models, mouse studies typically overestimate treatment benefit by 1.7-fold (range 1.2 to 2.5-fold) for efficacy endpoints (e.g., reduction in infarct size, improvement in tissue oxidative markers). Thermostable strains (ΔTm ≥ 10 °C) maintain 70–90% activity after 24 h, but wild-type mesophilic enzymes maintain <20% activity under the same conditions, according to studies using human serum [5,24]. These correction factors are provided as empirical guidelines, not as absolute predictors [24,70,71,108,110,112,113].

## 6. Challenges and Future Directions

### 6.1. The Development of Thermostable Therapeutic Enzymes

Thermostable therapeutic enzymes typically initiates at defined “weak spots” (surface loops, helix ends, β-sheet edges), while catalytic residues are located in spatially distinct regions. This segregation allows for targeted stabilization of unfolding-prone sites while preserving flexibility in the catalytic loops. However, the exclusive focus on thermostability has produced stable but therapeutically ineffective variants. Therefore, future engineering must adopt multi-objective optimization from the outset. Machine learning models (e.g., Gaussian processes, deep neural networks) trained on combined stability-activity datasets can identify mutations that enhance thermal resistance without compromising turnover rate.

### 6.2. Intracellular Delivery

For oxidative stress indications, particularly in neurodegenerative diseases, antioxidant enzymes need to reach intracellular compartments (cytosol, mitochondria). Thermostability does not confer membrane permeability. A promising advance is the development of thermostable “stealth” variants that retain their activity after cytosolic administration, avoiding rapid lysosomal degradation.

### 6.3. Scalability in Manufacturing

Thermostable enzymes offer inherent manufacturing advantages: simplified purification (thermal precipitation of host proteins), extended shelf life without cold chains, and resistance to shear stress. However, highly engineered variants with multiple disulfide bonds or non-canonical amino acids often express poorly in conventional *Escherichia coli* or yeast. Future directions include: (1) host engineering (strains with improved oxidative folding), (2) cell-free protein synthesis (bypassing toxicity and folding bottlenecks), and (3) continuous production platforms that integrate expression, purification, and formulation.

### 6.4. Translational Differences Between Species

Rodent models often overestimate the therapeutic efficacy of antioxidant enzymes because murine proteolytic environments are less aggressive than human serum and murine immune systems are more tolerant to foreign proteins.

## 7. Conclusions

Engineering thermostable antioxidant enzymes integrates structural biophysics and protein engineering, but there are several limitations that require caution. Thermostability stems from synergistic structural features (hydrophobic packing, salt bridges, disulfide bonds, rigid loops) and yet the optimal combination remains context-dependent and there is no universal solution.

Key challenges, at present, are represented by the stability-activity trade-off, immunogenicity of non-human scaffolds, inefficient intracellular delivery, scalability of production for disulfide-rich variants due to inefficient oxidative folding in conventional expression hosts, and species differences in proteolytic environments.

Regarding the activity-stability-selectivity trade-off, thermostability engineering can reduce both catalytic activity and substrate selectivity. Selectivity is preserved (≤2-fold change) in 85% of the designed variants but is eroded (>5-fold) in 15%, especially in the case of multiple cavity-filling mutations. Computational preselection reduces the loss of selectivity to <5%. For therapeutic applications, a ΔTm ≤ 15 °C generally preserves selectivity, while a ΔTm > 20 °C risks off-target effects.

In conclusion, thermostable antioxidant enzymes are structurally feasible and show promise preclinically. However, clinical validation remains the critical unmet step, and the remaining barriers are both biological and translational, as well as engineering-based.

## Figures and Tables

**Table 1:** Comparison of High-Throughput Screening Methods for Thermostability Engineering.

Method	Throughput (Variants/Day)	Temperature Range	Tm Accuracy	Success Rate	Key References
ThermoFAD	10,000–50,000	25–95 °C	±1.5 °C	72%	[36]
DSF (SYPRO Orange)	5000–20,000	20–100 °C	±1.0 °C	68%	[37,38,39]
CD spectroscopy	50–200	20–100 °C	±0.5 °C	85%	[40]
Differential scanning Fluorimetry	500–2000	20–110 °C	±0.3 °C	78%	[41,42,43]
Protease resistance assay	1000–10,000	37–60 °C	N/A	65%	[44]

**Table 2:** Computational Tools for Thermostability Prediction: Accuracy, Speed and Limitations.

Tool	Method	Limitations	Applications	Key References
FoldX	Empirical force field	No dynamics; limited water modeling	Rapid screening of single mutations	[49]
Rosetta (ddG_monomer)	Physics-based energy function	High computational cost; sampling limitations	High- confidence predictions with MD pre-filtering	[45,50,51]
Rosetta (Cartesian ddG)	All-atom with harmonic constraints	Very high computational cost	Critical mutations with experimental follow-up	[50,51]
FireProt	Consensus (FoldX + Rosetta + evolution)	Requires multiple sequence alignment	Combined stability-evolution predictions	[45]
PROSS	Rosetta + sequence design	Designed for multi-mutation combinations	Complete enzyme redesign	[23]
ABACUS	Statistical potential	Limited to soluble proteins	Preliminary screening	[45]
DeepDDG	Deep learning	Training bias toward single mutations	Large-scale mutation scanning	[52]
ThermoNet	Neural network	Requires retraining for new enzyme families	Family- specific predictions	[52]

**Table 3:** Engineering Human SOD1 for 15 °C Increased Tm Using Consensus Design and Disulfide Engineering.

Step	Methodology	Principal Conclusions	Supporting References
**Sequence alignment**	142 SOD1 homologues from mesophiles to hyperthermophiles (20–100 °C optima)	Identified 18 positions with >90% conservation in thermophiles but variable in mesophiles	[25,64]
**Consensus mutations**	Introduced 7 consensus residues (T35S, G41A, V42I, L84F, V118I, E121D, Q153R)	Tm increased from 58 °C → 68 °C; activity preserved at 94%	[15,63]
**Disulfide prediction**	Rosetta ddG + Disulfide by Design 2.0	Identified A4C/V7C pair (Cα distance 5.2 Å, χ3 = −87°)	[46,49,50,65]
**Disulfide engineering**	Introduced A4C/V7C disulfide bond	Tm 68 °C → 73 °C; no activity loss	[46,65,66]
**Combinatorial variant**	Combined consensus mutations + disulfide	Final T = 73 °C (+15 °C), t1/2 at 37 °C = 72 h (wild-type: 4 h)	[23,67]
**Protease resistance**	Trypsin challenge (0.1 mg/mL, 37 °C, 4 h)	91% residual activity vs. 12% for wild-type	[68,69]
**Therapeutic relevance**	Improved pharmacokinetics in murine model	Circulating half-life: 45 min → 8.5 h	[24,70,71]

**Table 4:** Quantitative contributions of structural determinants to enzyme thermostability across hierarchical levels.

Level	Determinant	Mechanism	Quantified Contribution (ΔTm, °C)	Key References
Primary	Proline frequency	Reduced backbone entropy	+0.5 to +2.0 per substitution	[46,52,65,66,67,68,75,76]
	Disulfide bonds	Entropic stabilization of unfolded state	+3 to +10 per bond	
	Charged residue content	Enhanced electrostatic interactions	+1 to +5	
Secondary	α-helix stabilization	Increased helical dipole moments	+2 to +8	[46,83,84,85]
	β-sheet edge protection	Reduced aggregation propensity	+3 to +6	
	Loop shortening	Decreased flexible regions	+1 to +4	
Tertiary	Hydrophobic core packing	Reduced internal cavities	+5 to +15	[23,75,76,83]
	Salt bridge networks	Cooperative electrostatic stabilization	+4 to +12	
	Aromatic-aromatic interactions	Edge-to-face stacking	+2 to +6	
Quaternary	Subunit interface strengthening	Increased oligomerization energy	+8 to +20	[25,84]

**Table 5:** Strategies for Simultaneous Improvement of Thermostability and Catalytic Activity.

Strategy	Mechanism	Activity	Key References
Improving substrate affinity	Enhancing enzyme-substrate binding through noncovalent interactions (hydrogen bonds, ionic interactions, hydrophobic contacts)	Increases catalytic efficiency even in stabilized enzymes	[5,21,80,81,90]
Optimizing electrostatic interactions	Rearranging charge-charge interactions on enzyme surface	Improves both thermostability and activity	[5,21,67,75,76]
Removing steric hindrances	Structure-guided engineering to enlarge catalytic pockets or remove hindrances	Improves substrate access without compromising stability	[21,73,80,81,91]
Active site flexibility modulation	Introducing appropriate mutations in or near the active site	Maintains or enhances activity while increasing overall rigidity	[21,67,80,81,92]
N- and C-terminal engineering	Truncation or stabilization of termini, including disulfide bridge introduction	Contributes to simultaneous improvement with minimal off-target effects	[5,21,46,65,93]
Enhanced hydrophobic interactions	Multiple mutations of hydrophobic residues in protein core	More significant than individual mutations; synergistic effects	[5,15,21,30,67]

**Table 6:** Representative preclinical studies of thermostable antioxidant enzymes for oxidative stress-related diseases.

Disease Model	Enzyme	Engineering Strategy	Animal Model	Outcomes	Supporting References
Myocardial I/R injury	SOD/catalase fusion	Thermophilic scaffold (*Thermus thermophilus*)	Rat	Infarct size ↓62%; half-life 8.5 h vs. 0.75 h	[24,70,71]
Parkinson’s disease	MnSOD	PEGylation + thermostable variant	Mouse	Striatal dopamine preservation 71%; motor function improved	[108,110]
Rheumatoid arthritis	Fe-SOD	Thermophilic Fe-SOD (*T. thermophilus*)	Rat adjuvant-induced	Joint swelling ↓58%; IL-1β ↓44%	[64,119,120]
Acute kidney injury	CuZnSOD	Consensus design + disulfide (Tm +15 °C)	Mouse ischemia–reperfusion	Serum creatinine ↓51%; tubular necrosis reduced	[89,93]
Acute lung injury	Catalase	Thermostable from *T. thermophilus*	Mouse LPS-induced	Neutrophil infiltration ↓65%; protein leak ↓70%	[112,113]
Diabetic complications	GPx mimic	Thermophilic scaffold (*P. furiosus* thioredoxin)	STZ-induced diabetic rat	Blood glucose not altered; oxidative markers ↓50%	[83,92,132]
Ischemic stroke	SOD	Disulfide-engineered human SOD1	Mouse MCAO	Infarct volume ↓55%; neurological score improved	[67,68]
Liver fibrosis	Catalase	Targeted to asialoglycoprotein receptor	CCl4-treated mouse	Fibrosis area ↓60%; α-SMA ↓55%	[114,115]

## Data Availability

No new data were created.

## Supplementary Materials

The following supporting information can be downloaded at: https://www.mdpi.com/article/10.3390/ijms27135695/s1.

## Abbreviations

The following abbreviations are used in this manuscript:

AQUI	Aqualysin I
FoldX	Computational Bioinformatics Tool
Taq	Thermus aquaticus
Gln	Glutamine
Asn	Asparagine
Cys	Cysteine
Met	Methionine
dNTPs	Deoxynucleoside Triphosphates
PCR	Polymerase Chain Reaction
StEP	Staggered Extension Process
FAD	Fluor-adapted
CD	Circular dichroism
Tm	Temperature
PTEs	Phosphotriesterases
ΔΔG	Delta Delta G
ML	Machine Learning
ROS	Reactive Oxygen Species
SOD	Superoxide dismutase
CAT	Catalase
GPx	Glutathione peroxidase
H2O2	Hydrogen peroxide
LDL	Low-density lipoproteine
ADA	Adenosine Deaminase
tES	Thermostable exoshells
DS-tES	Disulfide-linked tES
Nrf2	Nuclear factor erythroid 2
ATP	Adenosine triphosphate
TXA2	Thromboxane A2
VLPs	Virus-like particles extracellular vesicles
VEs	Extracellular vesicles

## References

[1] Singh R.S., Singh T., Pandey A. (2019). Microbial Enzymes—An Overview. Advances in Enzyme Technology.

[2] Singh R.S., Singh T., Singh A.K. (2019). Enzymes as Diagnostic Tools. Advances in Enzyme Technology.

[3] Cheung L.K.Y., Sanders A.D., Houfani A.A., Grahame D.A.S., Bryksa B.C., Dee D.R., Yada R.Y. (2024). Factors Affecting Enzyme Activity and Design. Improving and Tailoring Enzymes for Food Quality and Functionality.

[4] Thakur M., Tiwari S.K., Bansal S. (2025). L-Asparaginase: A Review of Microbial Production and Protein Engineering with Translational Applications for Oncology and Food Safety. World J. Microbiol. Biotechnol..

[5] Xu K., Fu H., Chen Q., Sun R., Li R., Zhao X., Zhou J., Wang X. (2025). Engineering Thermostability of Industrial Enzymes for Enhanced Application Performance. Int. J. Biol. Macromol..

[6] Khan M.F. (2025). Enhancing Stability of Enzymes for Industrial Applications: Molecular Insights and Emerging Approaches. World J. Microbiol. Biotechnol..

[7] Chisti Y. (2021). Chemical Stabilization of Enzymes. Enzymes.

[8] Michailidou F. (2023). Engineering of Therapeutic and Detoxifying Enzymes. Angew. Chem. Int. Ed..

[9] Lapuhs P., Fuhrmann G., Labrou N. (2019). Engineering Strategies for Oral Therapeutic Enzymes to Enhance Their Stability and Activity. Therapeutic Enzymes: Function and Clinical Implications.

[10] Pandey A., Dhakar K. (2026). Extreme Thermal Environments: Reservoirs of Industrially Important Thermozymes. Front. Microbiol..

[11] Anwar U.B., Zwar I.P., De Souza A.O. (2020). Biomolecules Produced by Extremophiles Microorganisms and Recent Discoveries. New and Future Developments in Microbial Biotechnology and Bioengineering.

[12] Bankar A., Patil S., Shinde M., Shinde S., Kowligi B. (2022). Potential of Microbial Extremophiles for Biotechnological Applications: An Overview. Microbial Extremozymes.

[13] Bajpai P. (2023). Industrial Applications of Thermophilic/Hyperthermophilic Enzymes. Developments and Applications of Enzymes from Thermophilic Microorganisms.

[14] Samanta D., Govil T., Saxena P., Thakur P., Narayanan A., Sani R.K. (2022). Extremozymes and Their Applications. Extremozymes and Their Industrial Applications.

[15] Pongsupasa V., Anuwan P., Maenpuen S., Wongnate T., Magnani F., Marabelli C., Paradisi F. (2022). Rational-Design Engineering to Improve Enzyme Thermostability. Enzyme Engineering.

[16] Óskarsson K.R., Sævarsson A.F., Kristjánsson M.M. (2020). Thermostabilization of VPR, a Kinetically Stable Cold Adapted Subtilase, via Multiple Proline Substitutions into Surface Loops. Sci. Rep..

[17] Che Hussian C.H.A., Leong W.Y. (2023). Thermostable Enzyme Research Advances: A Bibliometric Analysis. J. Genet. Eng. Biotechnol..

[18] Sillu D., Agnihotri C., Agnihotri S. (2022). Advances in Industrial Biocatalysis through Immobilized Extremozymes. Extremozymes and Their Industrial Applications.

[19] Beygmoradi A., Homaei A. (2017). Marine Microbes as a Valuable Resource for Brand New Industrial Biocatalysts. Biocatal. Agric. Biotechnol..

[20] Rajivgandhi G.N., Li W.-J. (2022). Biomedical Application of Marine Extremozymes. Microbial Extremozymes.

[21] Nezhad N.G., Rahman R.N.Z.R.A., Normi Y.M., Oslan S.N., Shariff F.M., Leow T.C. (2023). Recent Advances in Simultaneous Thermostability-Activity Improvement of Industrial Enzymes through Structure Modification. Int. J. Biol. Macromol..

[22] Thapa S., Li H., OHair J., Bhatti S., Chen F.-C., Nasr K.A., Johnson T., Zhou S. (2019). Biochemical Characteristics of Microbial Enzymes and Their Significance from Industrial Perspectives. Mol. Biotechnol..

[23] Shi J., Yuan B., Yang H., Sun Z. (2025). Recent Advances on Protein Engineering for Improved Stability. BioDesign Res..

[24] Ghosh S., Alam S., Rathore A.S., Khare S.K., Labrou N. (2019). Stability of Therapeutic Enzymes: Challenges and Recent Advances. Therapeutic Enzymes: Function and Clinical Implications.

[25] Xu S.-Y., Zhou L., Xu Y., Hong H.-Y., Dai C., Wang Y.-J., Zheng Y.-G. (2023). Recent advances in structure-based enzyme engineering for functional reconstruction. Biotechnol. Bioeng..

[26] Porter J.L., Rusli R.A., Ollis D.L. (2016). Directed Evolution of Enzymes for Industrial Biocatalysis. ChemBioChem.

[27] Cadet X.F., Gelly J.C., Van Noord A., Cadet F., Acevedo-Rocha C.G., Currin A., Swainston N. (2022). Learning Strategies in Protein Directed Evolution. Directed Evolution.

[28] Ye L., Yang C., Yu H. (2018). From Molecular Engineering to Process Engineering: Development of High-Throughput Screening Methods in Enzyme Directed Evolution. Appl. Microbiol. Biotechnol..

[29] Jeevanandam J., Tsenov G., Danquah M.K., Ruiz-Molena D., Boussios S., Ovsepian S.V. (2026). Smart Nanomedicines for Neurodegenerative Diseases: Empowering New Therapies with Molecular Imaging and Artificial Intelligence. Mol. Diagn. Ther..

[30] Maruoka S., Teramoto T., Watanabe K., Kakuta Y. (2025). Crystal Structure of a Family II Pyrophosphatase from *Thermodesulfobacterium commune* and Factors Enabling Its High-temperature Adaptation. FEBS Lett..

[31] Kotov V., Mlynek G., Vesper O., Pletzer M., Wald J., Teixeira-Duarte C.M., Celia H., Garcia-Alai M., Nussberger S., Buchanan S.K. (2021). In-Depth Interrogation of Protein Thermal Unfolding Data with MoltenProt. Protein Sci..

[32] Li X., Wang Y., Ma F., Zhao C., Zhang Y., Zhu Y., Zhang Y., Hou S., Li B., Yang F. (2025). Chaperone-Mediated Thermotolerance in Hyperthermophilic Composting: Molecular-Level Protein Remodeling of Microbial Communities. Environ. Sci. Ecotechnol..

[33] Kushwah V.C., Chauhan R., Dhaked R.K. (2024). Fluorescence Thermal Shift Assay Based High Through-Put Screening in Small Molecule Drug Discovery: A Brief Overview. J. Mod. Biol. Drug Discov..

[34] Mays Z.J., Mohan K., Trivedi V.D., Chappell T.C., Nair N.U. (2020). Directed Evolution of *Anabaena variabilis* Phenylalanine Ammonia-Lyase (PAL) Identifies Mutants with Enhanced Activities. Chem. Commun..

[35] Atsavapranee B., Stark C.D., Sunden F., Thompson S., Fordyce P.M. (2021). Fundamentals to Function: Quantitative and Scalable Approaches for Measuring Protein Stability. Cell Syst..

[36] Ito S., Matsunaga R., Nakakido M., Komura D., Katoh H., Ishikawa S., Tsumoto K. (2024). High-Throughput System for the Thermostability Analysis of Proteins. Protein Sci..

[37] Gao K., Oerlemans R., Groves M.R. (2020). Theory and Applications of Differential Scanning Fluorimetry in Early-Stage Drug Discovery. Biophys. Rev..

[38] Owens A.E., Iannotti M.J., Sanchez T.W., Voss T., Kapoor A., Hall M.D., Marugan J.J., Michael S., Southall N., Henderson M.J. (2022). High-Throughput Cellular Thermal Shift Assay Using Acoustic Transfer of Protein Lysates. ACS Chem. Biol..

[39] Bian J., Tan P., Nie T., Hong L., Yang G.-Y. (2024). Optimizing Enzyme Thermostability by Combining Multiple Mutations Using Protein Language Model. mLife.

[40] Miles A.J., Janes R.W., Wallace B.A. (2021). Tools and Methods for Circular Dichroism Spectroscopy of Proteins: A Tutorial Review. Chem. Soc. Rev..

[41] Nie M., Liu Y., Huang X., Zhang Z., Zhao Q. (2022). Microtiter Plate-Based Differential Scanning Fluorimetry: A High-Throughput Method for Efficient Formulation Development. J. Pharm. Sci..

[42] Lisina S., Inam W., Huhtala M., Howaili F., Zhang H., Rosenholm J.M. (2023). Nano Differential Scanning Fluorimetry as a Rapid Stability Assessment Tool in the Nanoformulation of Proteins. Pharmaceutics.

[43] Sun N., Xu Q., Yang M., Li Y., Liu H., Tantai W., Shu G., Li G. (2024). A High-Throughput Differential Scanning Fluorimetry Method for Rapid Detection of Thermal Stability and Iron Saturation in Lactoferrin. Int. J. Biol. Macromol..

[44] Li L., Liu X., Bai Y., Yao B., Luo H., Tu T. (2024). High-Throughput Screening Techniques for the Selection of Thermostable Enzymes. J. Agric. Food Chem..

[45] Planas-Iglesias J., Marques S.M., Pinto G.P., Musil M., Stourac J., Damborsky J., Bednar D. (2021). Computational Design of Enzymes for Biotechnological Applications. Biotechnol. Adv..

[46] Li G., Fang X., Su F., Chen Y., Xu L., Yan Y. (2018). Enhancing the Thermostability of Rhizomucor Miehei Lipase with a Limited Screening Library by Rational-Design Point Mutations and Disulfide Bonds. Appl. Environ. Microbiol..

[47] Han R.-Z., Xu G.-C., Dong J.-J., Ni Y. (2016). Arginine Deiminase: Recent Advances in Discovery, Crystal Structure, and Protein Engineering for Improved Properties as an Anti-Tumor Drug. Appl. Microbiol. Biotechnol..

[48] Childers M.C., Daggett V. (2017). Insights from Molecular Dynamics Simulations for Computational Protein Design. Mol. Syst. Des. Eng..

[49] Buß O., Rudat J., Ochsenreither K. (2018). FoldX as Protein Engineering Tool: Better Than Random Based Approaches?. Comput. Struct. Biotechnol. J..

[50] Sora V., Laspiur A.O., Degn K., Arnaudi M., Utichi M., Beltrame L., De Menezes D., Orlandi M., Stoltze U.K., Rigina O. (2023). RosettaDDGPrediction for High-throughput Mutational Scans: From Stability to Binding. Protein Sci..

[51] Huang P., Chu S.K.S., Frizzo H.N., Connolly M.P., Caster R.W., Siegel J.B. (2020). Evaluating Protein Engineering Thermostability Prediction Tools Using an Independently Generated Dataset. ACS Omega.

[52] Dou Z., Sun Y., Jiang X., Wu X., Li Y., Gong B., Wang L. (2023). Data-Driven Strategies for the Computational Design of Enzyme Thermal Stability: Trends, Perspectives, and Prospects. ABBS.

[53] Son A., Park J., Kim W., Yoon Y., Lee S., Park Y., Kim H. (2024). Revolutionizing Molecular Design for Innovative Therapeutic Applications through Artificial Intelligence. Molecules.

[54] Siedhoff N.E., Schwaneberg U., Davari M.D. (2020). Machine Learning-Assisted Enzyme Engineering. Methods in Enzymology.

[55] Esmaeilpour D., Hamblin M.R., Cheng J., Khosravi A., Liu J., Zarepour A., Zarrabi A., Sillanpää M., Nazarzadeh Zare E., Shen J. (2026). Artificial Intelligence Driven Protein Design and Sustainable Nanomedicine for Advanced Theranostics. Bioact. Mater..

[56] Medina-Ortiz D., Khalifeh A., Anvari-Kazemabad H., Davari M.D. (2025). Interpretable and Explainable Predictive Machine Learning Models for Data-Driven Protein Engineering. Biotechnol. Adv..

[57] Fatima Ali N., Khan S., Zahid S. (2025). A Critical Address to Advancements and Challenges in Computational Strategies for Structural Prediction of Protein in Recent Past. Comput. Biol. Chem..

[58] Hu Z., Liu Y., Huang Y., Yu P. (2026). Advances in the Directed Evolution of Computer-Aided Enzymes. CTMC.

[59] Ao Y., Dörr M., Menke M.J., Born S., Heuson E., Bornscheuer U.T. (2024). Data-Driven Protein Engineering for Improving Catalytic Activity and Selectivity. ChemBioChem.

[60] Cui X.-C., Zheng Y., Liu Y., Yuchi Z., Yuan Y.-J. (2025). AI-Driven de Novo Enzyme Design: Strategies, Applications, and Future Prospects. Biotechnol. Adv..

[61] Burton A.J., Thomson A.R., Dawson W.M., Brady R.L., Woolfson D.N. (2016). Installing Hydrolytic Activity into a Completely de Novo Protein Framework. Nat. Chem..

[62] Chia B.S., Seah Y.F.S., Wang B., Shen K., Srivastava D., Chew W.L. (2025). Engineering a New Generation of Gene Editors: Integrating Synthetic Biology and AI Innovations. ACS Synth. Biol..

[63] Korendovych I.V., Bornscheuer U.T., Höhne M. (2018). Rational and Semirational Protein Design. Protein Engineering.

[64] Ismaiel M.M.S., Piercey-Normore M.D. (2019). Molecular Characterization and Expression Analysis of Iron Superoxide Dismutase Gene from *Pseudochlorella pringsheimii* (Trebouxiophyceae, Chlorophyta). Physiol. Mol. Biol. Plants.

[65] Niu C., Zhu L., Xu X., Li Q. (2016). Rational Design of Disulfide Bonds Increases Thermostability of a Mesophilic 1,3-1,4-β-Glucanase from Bacillus Terquilensis. PLoS ONE.

[66] Xu R., Pan Q., Zhu G., Ye Y., Xin M., Wang Z., Wang S., Li W., Wei Y., Guo J. (2024). ThermoLink: Bridging Disulfide Bonds and Enzyme Thermostability through Database Construction and Machine Learning Prediction. Protein Sci..

[67] Siddiqui K.S. (2017). Defying the Activity–Stability Trade-off in Enzymes: Taking Advantage of Entropy to Enhance Activity and Thermostability. Crit. Rev. Biotechnol..

[68] Xie Y., Gu Y., Li Z., Zhang L., Hei Y. (2025). Effects of Exercise on Different Antioxidant Enzymes and Related Indicators: A Systematic Review and Meta-Analysis of Randomized Controlled Trials. Sci. Rep..

[69] Famutimi O.G., Adebiyi V.G., Akinmolu B.G., Dada O.V., Adewale I.O. (2024). Trypsin, Chymotrypsin and Elastase in Health and Disease. Future J. Pharm. Sci..

[70] Zaman R., Islam R.A., Ibnat N., Othman I., Zaini A., Lee C.Y., Chowdhury E.H. (2019). Current Strategies in Extending Half-Lives of Therapeutic Proteins. J. Control. Release.

[71] Pathivada K., Glassman P.M. (2025). Half-Life Extension of Therapeutics: Applications and Mechanisms. J. Pharmacol. Exp. Ther..

[72] Zhou J., Huang M. (2024). Navigating the Landscape of Enzyme Design: From Molecular Simulations to Machine Learning. Chem. Soc. Rev..

[73] Song Z., Zhang Q., Wu W., Pu Z., Yu H. (2023). Rational Design of Enzyme Activity and Enantioselectivity. Front. Bioeng. Biotechnol..

[74] Baykov A.A., Anashkin V.A., Salminen A., Lahti R. (2017). Inorganic Pyrophosphatases of Family II—Two Decades after Their Discovery. FEBS Lett..

[75] Sucharski F., Gallo G., Coelho C., Hardy L., Würtele M. (2023). Modeling the Role of Charged Residues in Thermophilic Proteins by Rotamer and Dynamic Cross Correlation Analysis. J. Mol. Model..

[76] Shibuya A., Yokote M., Suzuki A., Fukui K., Yano T. (2024). An Extensive Ion-Pair/Hydrogen-Bond Network Contributes to the Thermostability of the MutL ATPase Domain from *Aquifex aeolicus*. FEMS Microbiol. Lett..

[77] Fukui K., Kondo N., Murakawa T., Baba S., Kumasaka T., Yano T. (2024). dUTP Pyrophosphatases from Hyperthermophilic Eubacterium and Archaeon: Structural and Functional Examinations on the Suitability for PCR Application. Protein Sci..

[78] Zeiske T., Stafford K.A., Palmer A.G. (2016). Thermostability of Enzymes from Molecular Dynamics Simulations. J. Chem. Theory Comput..

[79] Braun M., Tripp A., Chakatok M., Kaltenbrunner S., Fischer C., Stoll D., Bijelic A., Elaily W., Totaro M.G., Moser M. (2026). Computational Enzyme Design by Catalytic Motif Scaffolding. Nature.

[80] Mu D., Wang D., Montalbán-López M., Wu X., Li X. (2025). Enhancement of Thermostability and Catalytic Activity of β-1,4-Xylanase via Rational and Semi-Rational Collaborative Engineering. J. Agric. Food Chem..

[81] Yu X., Hu Y., Li Q., Lv Y., Tang H., Wen L., Cheng Y., Chen Z., Zhang T., Wu H. (2025). Overview of Various Protein Engineering Strategies to Improve the Catalytic Activity, Thermostability, and Acid/Base Stability of β-Glucanase. Int. J. Biol. Macromol..

[82] Sadeghi S., Vallerinteavide Mavelli G., Vaidya S.S., Drum C.L. (2022). Gastrointestinal Tract Stabilized Protein Delivery Using Disulfide Thermostable Exoshell System. Int. J. Mol. Sci..

[83] Chang J., Zhang C., Cheng H., Tan Y.-W. (2021). Rational Design of Adenylate Kinase Thermostability through Coevolution and Sequence Divergence Analysis. Int. J. Mol. Sci..

[84] Finch A.J., Kim J.R. (2018). Thermophilic Proteins as Versatile Scaffolds for Protein Engineering. Microorganisms.

[85] Koroleva V., Lavlinskaya M., Holyavka M., Penkov N., Zuev Y., Artyukhov V. (2024). Thermal Inactivation, Denaturation and Aggregation Processes of Papain-Like Proteases. Chem. Biodivers..

[86] Yang J., Deng B., Liao P., Lin S., Zheng L., Yang X., Wang F., Zhai C., Ma L. (2025). Improving the Catalytic Activity and Thermostability of FAST-PETase with a Multifunctional Short Peptide. Biomolecules.

[87] Suriya J., Bharathiraja S., Krishnan M., Manivasagan P., Kim S.-K. (2016). Extremozymes from Marine Actinobacteria. Advances in Food and Nutrition Research.

[88] Haddadzadegan S., Dorkoosh F., Bernkop-Schnürch A. (2022). Oral Delivery of Therapeutic Peptides and Proteins: Technology Landscape of Lipid-Based Nanocarriers. Adv. Drug Deliv. Rev..

[89] Hu J., Yu K., Jiang S., You G., Lin W., Wang Q., Wang A., Pei X. (2025). Site-Directed Immobilization of Enzymes on Microporous Resins Depended on Transglutaminase-Catalyzed Bioconjugation. Int. J. Biol. Macromol..

[90] Han X., Wang Y., Wei X., Yu H., Liu S., Tan H., Xin F. (2026). Improving the Catalytic Efficiency of a Highly Thermostable Phenylalanine Ammonia-Lyase from *Nostoc* sp. ATCC 53789 and Its Application in Producing Low L-Phenylalanine Protein. Food Chem..

[91] Reetz M. (2022). Making Enzymes Suitable for Organic Chemistry by Rational Protein Design. ChemBioChem.

[92] Nguyen K., Lee C. (2025). Catalytic His-Loop Flexibility Drives High Activity in Hyperthermophilic Esterase EstE1 While Preserving Structural Stability. Microbiol. Spectr..

[93] Ding N., Jiang Y., Lee S., Cheng Z., Ran X., Ding Y., Ge R., Zhang Y., Yang Z.J. (2025). Enzyme Miniaturization: Revolutionizing Future Biocatalysts. Biotechnol. Adv..

[94] Amrein B.A., Steffen-Munsberg F., Szeler I., Purg M., Kulkarni Y., Kamerlin S.C.L. (2017). *CADEE*: Computer-Aided Directed Evolution of Enzymes. IUCrJ.

[95] Vivek K., Sandhia G.S., Subramaniyan S. (2022). Extremophilic Lipases for Industrial Applications: A General Review. Biotechnol. Adv..

[96] Drucker D.J. (2020). Advances in Oral Peptide Therapeutics. Nat. Rev. Drug Discov..

[97] Zhu Q., Chen Z., Paul P.K., Lu Y., Wu W., Qi J. (2021). Oral Delivery of Proteins and Peptides: Challenges, Status Quo and Future Perspectives. Acta Pharm. Sin. B.

[98] Calzini M.A., Malico A.A., Mitchler M.M., Williams G.J. (2021). Protein Engineering for Natural Product Biosynthesis and Synthetic Biology Applications. Protein Eng. Des. Sel..

[99] Jimenez-Rosales A., Flores-Merino M.V. (2018). Tailoring Proteins to Re-Evolve Nature: A Short Review. Mol. Biotechnol..

[100] Wu J., Wang Z., Zeng M., He Z., Chen Q., Chen J. (2024). Comprehensive Understanding of Laboratory Evolution for Food Enzymes: From Design to Screening Innovations. J. Agric. Food Chem..

[101] Pai P.P., Mondal S. (2017). Applying Knowledge of Enzyme Biochemistry to the Prediction of Functional Sites for Aiding Drug Discovery. CTMC.

[102] Mozaffari S., Moen A., Ng C.Y., Nicolaes G.A.F., Wichapong K. (2025). Structural Bioinformatics for Rational Drug Design. Res. Pract. Thromb. Haemost..

[103] Talevi A., Gore M., Jagtap U.B. (2024). Computer-Aided Drug Discovery and Design: Recent Advances and Future Prospects. Computational Drug Discovery and Design.

[104] Agarwal U., Tonk R.K., Paliwal S. (2025). Importance of Computer-Aided Drug Design in Modern Pharmaceutical Research. Curr. Drug Discov. Technol..

[105] Afzal S., Abdul Manap A.S., Attiq A., Albokhadaim I., Kandeel M., Alhojaily S.M. (2023). From Imbalance to Impairment: The Central Role of Reactive Oxygen Species in Oxidative Stress-Induced Disorders and Therapeutic Exploration. Front. Pharmacol..

[106] Jomova K., Alomar S.Y., Alwasel S.H., Nepovimova E., Kuca K., Valko M. (2024). Several Lines of Antioxidant Defense against Oxidative Stress: Antioxidant Enzymes, Nanomaterials with Multiple Enzyme-Mimicking Activities, and Low-Molecular-Weight Antioxidants. Arch. Toxicol..

[107] Krishnamurthy H.K., Rajavelu I., Pereira M., Jayaraman V., Krishna K., Wang T., Bei K., Rajasekaran J.J. (2024). Inside the Genome: Understanding Genetic Influences on Oxidative Stress. Front. Genet..

[108] Cecerska-Heryć E., Surowska O., Heryć R., Serwin N., Napiontek-Balińska S., Dołęgowska B. (2021). Are Antioxidant Enzymes Essential Markers in the Diagnosis and Monitoring of Cancer Patients—A Review. Clin. Biochem..

[109] Sebghatollahi Z., Yogesh R., Mahato N., Kumar V., Mohanta Y.K., Baek K.-H., Mishra A.K. (2025). Signaling Pathways in Oxidative Stress-Induced Neurodegenerative Diseases: A Review of Phytochemical Therapeutic Interventions. Antioxidants.

[110] Goel F., Garg V.K. (2026). Noni (*Morinda citrifolia*) and Neurodegeneration: Exploring Its Role in Oxidative Stress, Neuroinflammation, and Cell Survival Pathways. Mol. Neurobiol..

[111] Aldrich J.L., Panicker A., Ovalle R., Sharma B. (2023). Drug Delivery Strategies and Nanozyme Technologies to Overcome Limitations for Targeting Oxidative Stress in Osteoarthritis. Pharmaceuticals.

[112] Liu H., Dong J., Xu C., Ni Y., Ye Z., Sun Z., Fan H., Chen Y. (2025). Acute Lung Injury: Pathogenesis and Treatment. J. Transl. Med..

[113] Bezerra F.S., Lanzetti M., Nesi R.T., Nagato A.C., Silva C.P.E., Kennedy-Feitosa E., Melo A.C., Cattani-Cavalieri I., Porto L.C., Valenca S.S. (2023). Oxidative Stress and Inflammation in Acute and Chronic Lung Injuries. Antioxidants.

[114] Rani R., Gandhi C.R. (2023). Stellate cell in hepatic inflammation and acute injury. J. Cell. Physiol..

[115] Kumar S., Duan Q., Wu R., Harris E.N., Su Q. (2021). Pathophysiological Communication between Hepatocytes and Non-Parenchymal Cells in Liver Injury from NAFLD to Liver Fibrosis. Adv. Drug Deliv. Rev..

[116] Virk T.L., Liu Q., Yuan Y., Xu X., Chen F. (2025). Curcumin as Therapeutic Modulator of Impaired Antioxidant Defense System: Implications for Oxidative Stress-Associated Reproductive Dysfunction. Biology.

[117] Liu H., Yu R., Gao Y., Li X., Guan X., Thomas K., Xiu M., Zhang X. (2022). Antioxidant Enzymes and Weight Gain in Drug-Naive First-Episode Schizophrenia Patients Treated with Risperidone for 12 Weeks: A Prospective Longitudinal Study. Curr. Neuropharmacol..

[118] Zhang M., Hu W., Cai C., Wu Y., Li J., Dong S. (2022). Advanced Application of Stimuli-Responsive Drug Delivery System for Inflammatory Arthritis Treatment. Mater. Today Bio.

[119] Al-Adeeb A., Aqeel S.M., Aljaberi H.S., Gu Q., Jiang S., Ma S., Yu X. (2025). Advancing Cu/Zn Superoxide Dismutase (SOD1) Production in *Pichia pastoris*: Challenges, Strategies, Current Research Status, and Future Directions. Prep. Biochem. Biotechnol..

[120] Grujicic J., Allen A.R. (2025). Manganese Superoxide Dismutase: Structure, Function, and Implications in Human Disease. Antioxidants.

[121] Thao N.T.M., Do H.D.K., Nam N.N., Tran N.K.S., Dan T.T., Trinh K.T.L. (2023). Antioxidant Nanozymes: Mechanisms, Activity Manipulation, and Applications. Micromachines.

[122] Deshwal A., Tripathi R.M., Saxena K., Sheikh F.A., Mishra P. (2024). Auriferous Nanozymes: Advances in Diagnostic and Therapeutic Applications. Nanotechnology.

[123] Raghavendra T., Bhat S.G. (2022). Enzyme Immobilized Nanomaterials. Nanomaterials for Biocatalysis.

[124] Delsuc N. (2023). Engineering Peptidyl and Protein Glutathione Peroxidase Mimics. Peptide and Protein Engineering for Biotechnological and Therapeutic Applications.

[125] Elsässer B., Zauner F.B., Messner J., Soh W.T., Dall E., Brandstetter H. (2017). Distinct Roles of Catalytic Cysteine and Histidine in the Protease and Ligase Mechanisms of Human Legumain As Revealed by DFT-Based QM/MM Simulations. ACS Catal..

[126] Özdemir G., Dede B. (2025). Multi-Enzyme Mimicry by Dinuclear Transition Metal Complexes with Oxime Ligand. J. Adv. Res. Nat. Appl. Sci..

[127] Demkiv O., Stasyuk N., Serkiz R., Gayda G., Nisnevitch M., Gonchar M. (2021). Peroxidase-Like Metal-Based Nanozymes: Synthesis, Catalytic Properties, and Analytical Application. Appl. Sci..

[128] Ben Hadj Hammouda Y., Coulibaly K., Bathily A., Teoh Sook Han M., Policar C., Delsuc N. (2022). Improvement of Peptidyl Copper Complexes Mimicking Catalase: A Subtle Balance between Thermodynamic Stability and Resistance towards H_2_O_2_ Degradation. Molecules.

[129] Bilal M., Khaliq N., Ashraf M., Hussain N., Baqar Z., Zdarta J., Jesionowski T., Iqbal H.M.N. (2023). Enzyme Mimic Nanomaterials as Nanozymes with Catalytic Attributes. Colloids Surf. B Biointerfaces.

[130] Ren X., Chen D., Wang Y., Li H., Zhang Y., Chen H., Li X., Huo M. (2022). Nanozymes-Recent Development and Biomedical Applications. J. Nanobiotechnol..

[131] Wang P., Min D., Chen G., Li M., Tong L., Cao Y. (2021). Inorganic Nanozymes: Prospects for Disease Treatments and Detection Applications. Front. Chem..

[132] Spagnoli G., Bolchi A., Cavazzini D., Pouyanfard S., Müller M., Ottonello S. (2017). Secretory Production of Designed Multipeptides Displayed on a Thermostable Bacterial Thioredoxin Scaffold in Pichia Pastoris. Protein Expr. Purif..

[133] Guo R., Du Q., He Y., Wu H., Zhang Y., Jing Z. (2026). Innovative Strategies to Overcome Stability Challenges of Single-Atom Nanozymes. Nano-Micro Lett..

[134] Fadeel B. (2024). Nanomaterials as Protein Mimics or Nanologicals. Nanomedicine.

[135] Feng Y., Chen J., Ning C. (2025). Advances in Biomedical Applications of Se-Based Nanozymes. J. Nanobiotechnol..

[136] Xiao N., Li Y., Sun P., Zhu P., Wang H., Wu Y., Bai M., Li A., Ming W. (2024). A Comparative Review: Biological Safety and Sustainability of Metal Nanomaterials Without and with Machine Learning Assistance. Micromachines.

