# Supplementary material for: Thermostability Engineering in Therapeutic Antioxidant Enzymes: From Molecular Fundamentals to Oxidative Stress Applications
Source: Int J Mol Sci. 2026 Jun 24;27(13):5695. doi: 10.3390/ijms27135695 (PMC13361086; doi:10.3390/ijms27135695)
Supplement: Supplementary file 1 [file ijms-27-05695-s001.zip › ijms-4323760-supplementary.pdf]

Supplementary File S1

Keywords and Subject Headings Used During the Search

**Database**	**Search strategy**
PubMed accessed 9 March, 2026	("Protein Engineering"[Mesh] OR "Directed Molecular Evolution"[Mesh] OR "Enzymes"[Mesh] OR "Biocatalysis"[Mesh])<br>AND<br>("Thermodynamics"[Mesh] OR "thermal stability"[tiab] OR "heat stable"OR "engineered enzyme*")<br>AND<br>("Systemic Inflammatory Response Syndrome"[Mesh] OR "Diabetes Mellitus"[Mesh] OR "Cardiovascular Diseases"[Mesh] OR "systemic")
Scopus accessed 9 March, 2026	("protein engineering" OR "enzyme engineering" OR "directed evolution" OR "rational design" OR "computational design" OR biocatalys*)<br>AND<br>(thermostab* OR "thermal stability" OR "heat stable" OR "thermally stable" OR "kinetic stability")<br>AND<br>("systemic disease" OR atherosclerosis OR diabetes OR "cardiovascular" OR inflammation OR "inflammatory mediator")
Web Of Science accessed 9 March, 2026	("enzyme engineering" OR "protein design" OR "directed evolution" OR "semi-rational design" OR "computational mutagenesis")<br>AND<br>("thermostability" OR "thermal stabilization" OR "Tm increase" OR "half-life" OR "proteolytic resistance")<br>AND<br>("oral-systemic" OR "periodontitis-systemic" OR "periodontal medicine" OR "gingipain" OR "oral pathogen*")<br>AND<br>("systemic inflammation" OR "cardiometabolic" OR "diabetes complication*" OR "bacteremia" OR "virulence factor")
Embase accessed 9 March, 2026	protein engineering'/exp OR 'directed evolution'/exp OR 'enzyme'/exp OR 'biocatalysis'/exp<br>AND<br>'thermostability'/exp OR 'protein stability'/exp<br>AND<br>'systemic disease'/exp OR 'inflammation'/exp OR 'diabetes mellitus'/exp OR 'cardiovascular disease'/exp<br>AND<br>(thermostab* OR 'heat stable' OR 'engineered enzyme*')
